# “Tonic Treatment” of Syphilis*Read before the Buffalo Medical Club, Sept. 3d, 1879.

**Published:** 1880-02

**Authors:** P. W. Van Peyma


### "TONIC TREATMENT" OF SYPHILIS.*

BY P. W. VAN PEYMA, M. D.

THE purpose of this paper is to direct attention to the so-called "Tonic Treatment of Syphilis," as advanced more particularly by Keyes, of New York, which, since the time of its introduction to the general notice of the profession, has received the approval of many medical men of high standing. It is not, therefore, my intention to review the history of the treatment of syphilis. This has undergone many radical changes since the time that this disease first attracted general attention, some centuries ago. The pendulum has swung from one extreme to the other, and back again a number of times; especially is this true in regard to the employment of mercury as a curative agent—many times denounced as entirely wanting in usefulness and even as terrible in its evil effects, and again extolled as a specific as valuable as it is harmless. I shall confine myself to a review of the points brought forward by Keyes, in a little work entitled "The Tonic Treatment of Syphilis."

Keyes in this preface of the work introduces the subject as follows:

"My studies in syphilitic blood have yielded results at once so gratifying to me, and so convincing as to the tonic influence of minute doses of mercury, that I feel impelled to lay this brief treatise before the medical public in support of a continuous treatment of syphilis by small (tonic) doses of mercury." Then again in the first chapter he says, "a rational treatment of syphilis must rest upon a surer foundation than the mere statement of its value made by him who employs it. Such a statement has been made again and again, by different people employing the most varied means against the same evil, and, with apparent justification, for it is well known that a majority of the most visible lesions of syphilis (the contaneous efflorescences) tend to subside spontaneously, and thus, as Founier happily expressed it, "to afford a triumph to every mode of treatment."

* Read before the Buffalo Medical Club, Sept. 3d, 1879.

After reviewing the various modes of treatment, he proceeds to the subject proper—the treatment of syphilis by small doses of mercury long continued. After quoting a number of authorities tending to show that mercury has frequently been considered a tonic in the treatment of chronic diseases generally, he adduces his principal proof of its tonic effect by what is really to be considered as a scientific demonstration. As a result of numerous microscopical examinations of blood, syphilitic and healthy, and as modified by the taking of mercury in large and small doses, Keyes and a number of others have proved that, first, syphilitic blood contains far less red blood corpuscles than healthy blood; second, that small doses, say one or two centigrammes of the protoiodide thrice daily, increase the amount when the number is abnormally diminished as a result of the syphilitic poison or otherwise; and thirdly, that large doses have the opposite effect, diminishing the number materially. These facts he determines by means of a hematimetre, which is simply an arrangement for dividing the field of the microscope into numerous small spaces, thus facilitating the counting of the blood corpuscles. The blood is diluted according to a definite proportion. This is done by means of a fluid obtained from human urine, prepared as follows: "Take of urine, neutral or slightly alkaline, sp. gr. 1020, a sufficient quantity, filtered, add gr. v of cor. sub. in powder for each ounce of urine.

"This will throw down dense clouds of amorphous urates, so fine that ordinary filter paper will not remove them. After standing, the urates deposit, and the clear fluid above may be easily decanted. Reduce with water sp. gr. 1020. The result is a limpid sparkling acid fluid which remains clear, no matter how often contaminated with the pipette, and does not seem to allow the growth of any form of vegetation. It makes a perfect mixture with blood." (See page 24 and 25).

The plan of treatment is then to commence with a small dose, say one centigramme, three times a day, continue this three days, then increase one centigramme, given two or three times daily for about the same length of time. This rate of increase is continued until its effect on the gums begins to show itself; we have then reached the full dose, so called, which varies considerably in different persons. Half of this dose, called the tonic dose, is then given for a period of three years. The most convenient mode of administration is by means of centigramme granules, taking one, two or three, or more, as the case may be. During the course of treatment should there be an outbreak of symptoms, it is advised, either to increase at once to the full dose, or what is probably preferable, supplement the tonic dose with inunctions or fumigations. As to the period of commencing treatment it is recommended to begin as soon as the diagnosis is positively established. This may not be perhaps until the rash or sore throat has manifested itself. That the diagnosis should be thus positively established is evident, as otherwise the patient might for the rest of his life-time be the subject of well grounded doubt and fear—a most unpleasant mental condition.

Under this treatment it is claimed that patients, with rare exceptions, will enjoy their usual health, and in some instances, profess to unusual freedom from aches and pains, and other morbid symptoms. The blood corpuscles increase in number rapidly, the previous pallor gives way to a healthy color,and life looks bright once more.

Under this treatment Dr. Keyes has failed to see any returning manifestations of disease in patients after ten years of unintermittent health. These have married and have had healthy children to all appearances. This is the only medical treatment recommended, except that in certain cases a short course of iodide of potass. may sometimes be advisable. I may say here that during the last year I have treated half a dozen cases according to this plan and am very much pleased with the result. In two instances the patients have as yet had no secondary symptoms, with the exception of a very slight sore throat, and this after more than eight times the usual period of incubation has gone by. They apparently enjoy perfect health. In the other instances the symptoms rapidly disappeared and there has been no return; on the contrary, the patients appear strong and well.

In the *Maryland Medical Jourual* for July, the leading article is one upon some observations on the treatment of syphilis, by J. Shelton Hill, M. D., read before the Baltimore Medical Association, in March, 1879. As a historical review of the past treatment of this disease, it is quite exhaustive, but it was written principally with a view to giving the author's opinion and conclusions regarding this particular plan of treatment.

His conclusions, based upon a considerable number of cases extending over years, is confirmatory of Dr. Keyes' observations.

His microscopical examinations of blood likewise agree with and confirm those of Dr. Keyes. If the conclusions arrived at by these observers became established we shall have additional testimony of the value of scientific experimentation in general and microscopical observation in particular.

In conclusion I wish to urge a fair trial of this method of treatment. If, as Dr. Keyes and others believe, that, by the long continued use of small doses of the protoiodide of mercury a complete eradication of the syphilitic poison may be effected, it is very important that the fact be generally known and acted upon. The method is simple, and judging from existing evidence, it promises much.

